# Supplementary material for: Exploring TAS2R46 biomechanics through molecular dynamics and network analysis
Source: Front Mol Biosci. 2024 Dec 2;11:1473675. doi: 10.3389/fmolb.2024.1473675 (PMC11646861; doi:10.3389/fmolb.2024.1473675)
Supplement: Supplementary file 1 [file DataSheet1.pdf]

Supplementary Material

1 Supplementary Methods

As TAS2Rs have been considered to be class A GPCR until human TAS2R46 structure was resolved, the A^{100} activity indicator, specifically developed to describe class A GPCR activation, was computed as reported in previous literature (Ibrahim et al., 2019; Calderón et al., 2023). In particular, the A^{100} is computed as:

$$A^{100} = -14.43\ R^{I27,W281} - 7.62\ R^{R55,H93} + 9.11\ R^{L98,S129} - 6.32\ R^{C203,H228} - 5.22\ R^{S252,F261} + 278.88$$

where R stands for the distance between the alpha carbons of the residues given in the superscript.

Moreover, as the outward movement of the TM6 IC region is one of the hallmarks of the class A GPCRs activation process (Zhou et al., 2019), the same conformational change was characterized. In particular, the distance between the IC areas of TM3 and TM6 in the three states was computed as the distance between the centre of mass of residues 104 to 100 (TM3) and the centre of mass of residues 218 to 224 (TM6).

2 Supplementary Figures

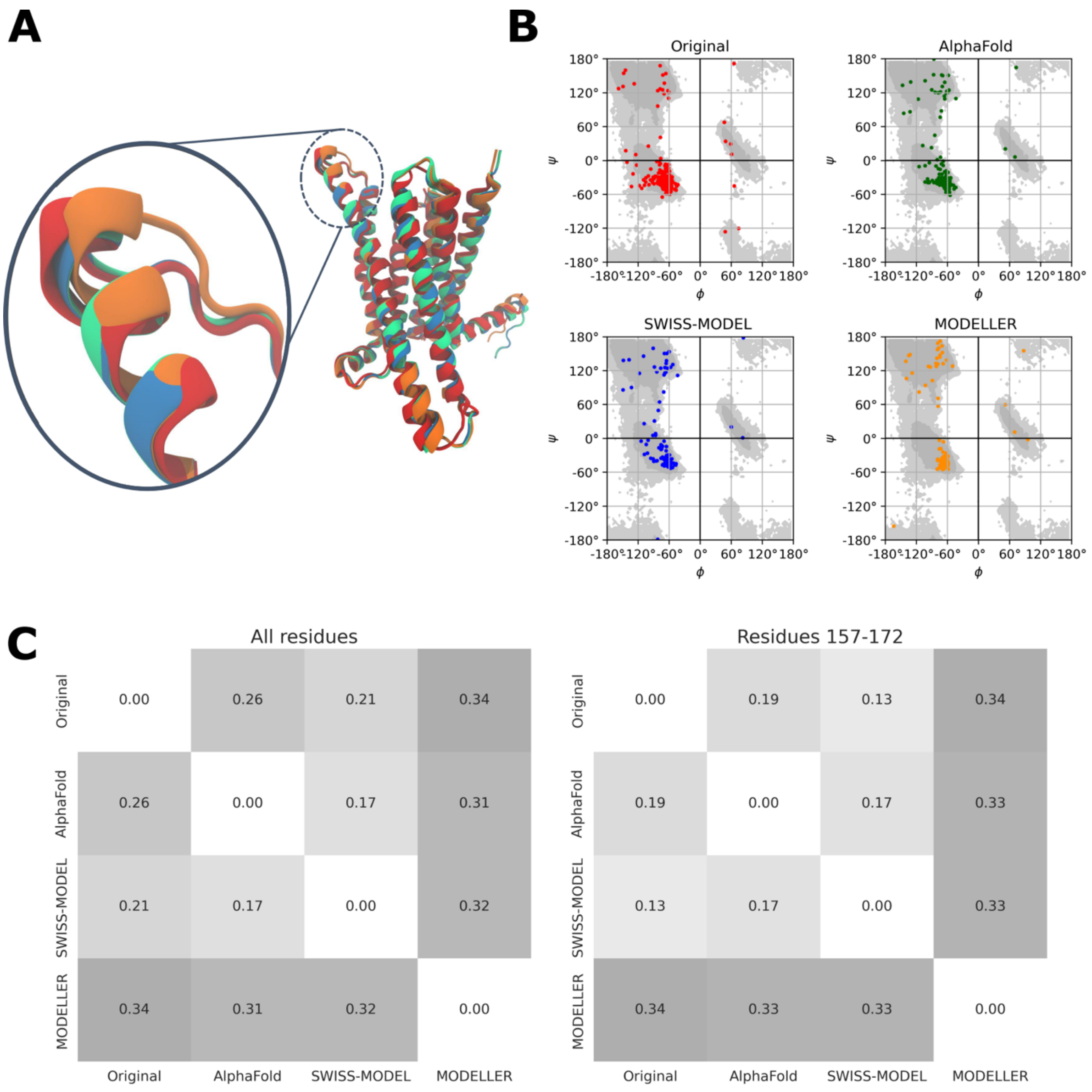

Supplementary Figure 1. (A) Molecular models of the human TAS2R46. The model used in the present work is represented in red, the AlphaFold model in green, the SWISS-MODEL in blue and the MODELLER model in orange. Residues 157-172, belonging to the ECL2 and missing in the experimental structure (PDB: 7XP6), are also represented enlarged. (B) Ramachandran plots of the molecular models of the human TAS2R46. The model investigated in the present study (referred to as "Original") is represented in red, the AlphaFold model in green, the SWISS-MODEL model in blue and the MODELLER model in orange. (C) RMSD matrices between the molecular models of TAS2R46. Numbers are in the nm unit. On the left, RMSD has been calculated using the positions of all atoms (excluding hydrogen atoms), whereas, on the right, the RMSD was evaluated only on residues 157-172 which are missing in the experimental structure (PDB: 7XP6). Darker values represent higher values of RMSD.

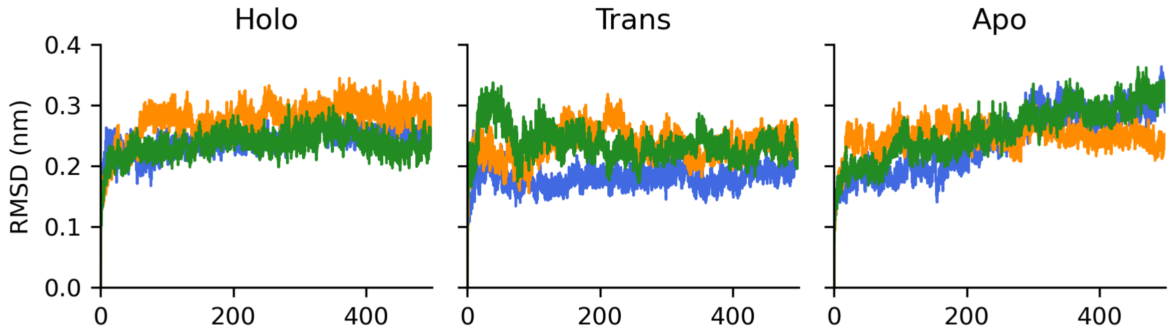

Supplementary Figure 2. RMSD of backbone atoms with respect to the initial conformation of the MD simulations for the Holo, Trans, and Apo states. The three replicas are displayed in different colours.

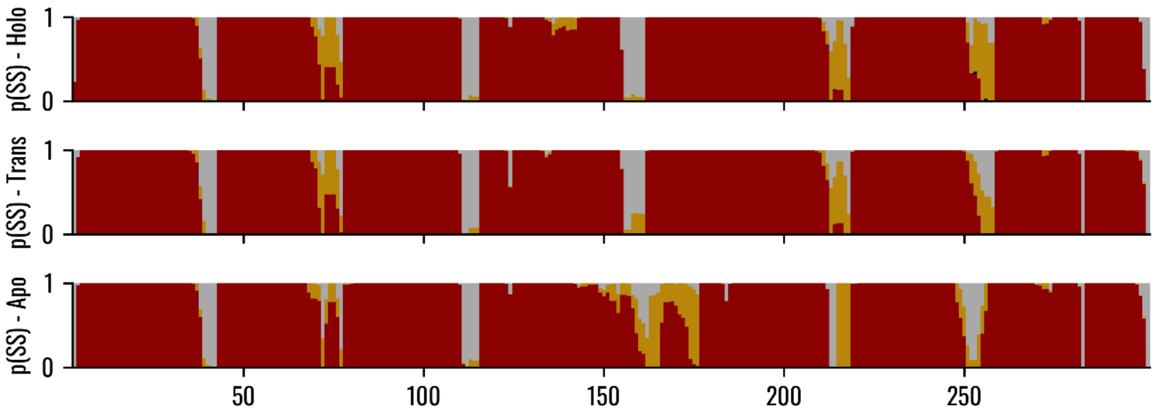

Supplementary Figure 3. Probability of each TAS2R46 residue to be involved in the formation of a secondary structure. The secondary structure probability was determined by predicting the secondary structure for each frame of the simulation. For each residue, the occurrences of different secondary structure conformations (helices, beta-sheets, turns, and coils) were counted. The total count for each structure type was then divided by the total number of frames, yielding the probability of a specific secondary structure for each residue. Helices are reported in dark red, beta sheets in dark blue, turns in dark yellow, and coils in grey.

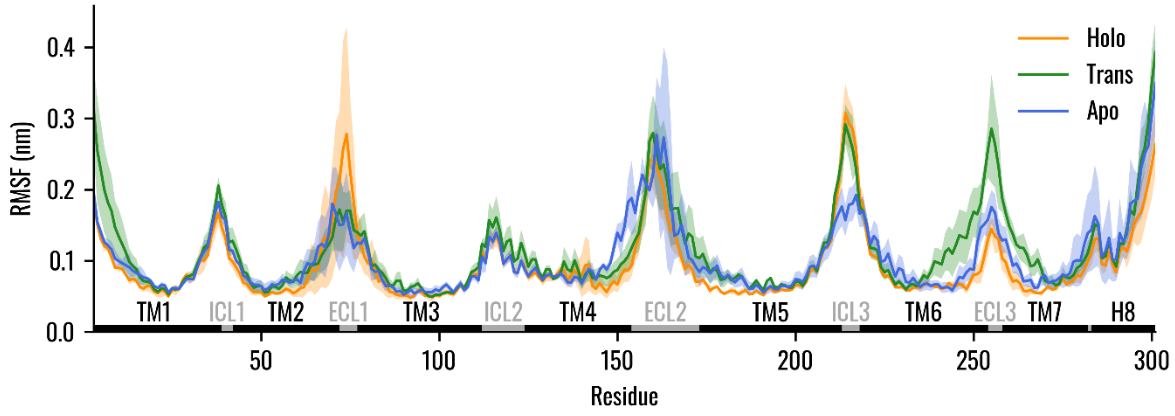

Supplementary Figure 4. RMSF of alpha carbons computed on the equilibrium trajectories (last 400 ns). The mean value across the three replicas is represented with a continuous line, whereas the shaded regions represent the standard deviation from the mean.

2.1 Cluster Analysis

The most frequent structural rearrangements of the three systems were also analysed with a cluster analysis (Supplementary Figure 5). Specifically, we analysed the concatenated final 400 ns of each replica, sampled at a 1 ns time step, using the linkage clustering algorithm implemented in GROMACS. The analysis was performed with an RMSD cutoff of 0.1 nm, considering the backbone atoms. The cluster analysis revealed 22 clusters for the Holo state and 12 clusters for the Apo state. In contrast, the Trans system exhibited 105 clusters, indicating greater structural fluctuations compared to the other states. Considering only clusters accounting for more than 90% of structures (i.e. simulation frames), the number of clusters decreased to 4, 12, and 3 for Holo, Trans and Apo states, respectively (Supplementary Figure 5A, B). For these clusters, we also evaluated the average per-residue RMSD. Specifically, for each centroid, we calculated the RMSD of each residue compared to the centroid of the most populated cluster in the Holo state. We then obtained the average per-residue RMSD for each system by averaging the RMSD values across the centroids of the system. This approach allowed us to highlight the main structural deviations of each residue among the systems' clusters compared to the chosen reference structure (see Supplementary Figure 3D, E). The Holo and Trans states demonstrated only minor differences, with ECL3 showing higher variability among the cluster centroids in both systems. In contrast, the Apo state, as expected due to the remarkable differences in the starting experimental structures, exhibited high deviations in its most frequent conformational states compared to the Holo reference. The largest variations were observed in the loop regions (ECL1, ECL2, ICL3, and ECL3), but even the structured transmembrane helices showed different rearrangements compared to the Holo structure. Notably, TM3 and ICL2 displayed only minor differences among the three systems, indicating their structural stability regardless of the receptor state or the presence of the agonist.

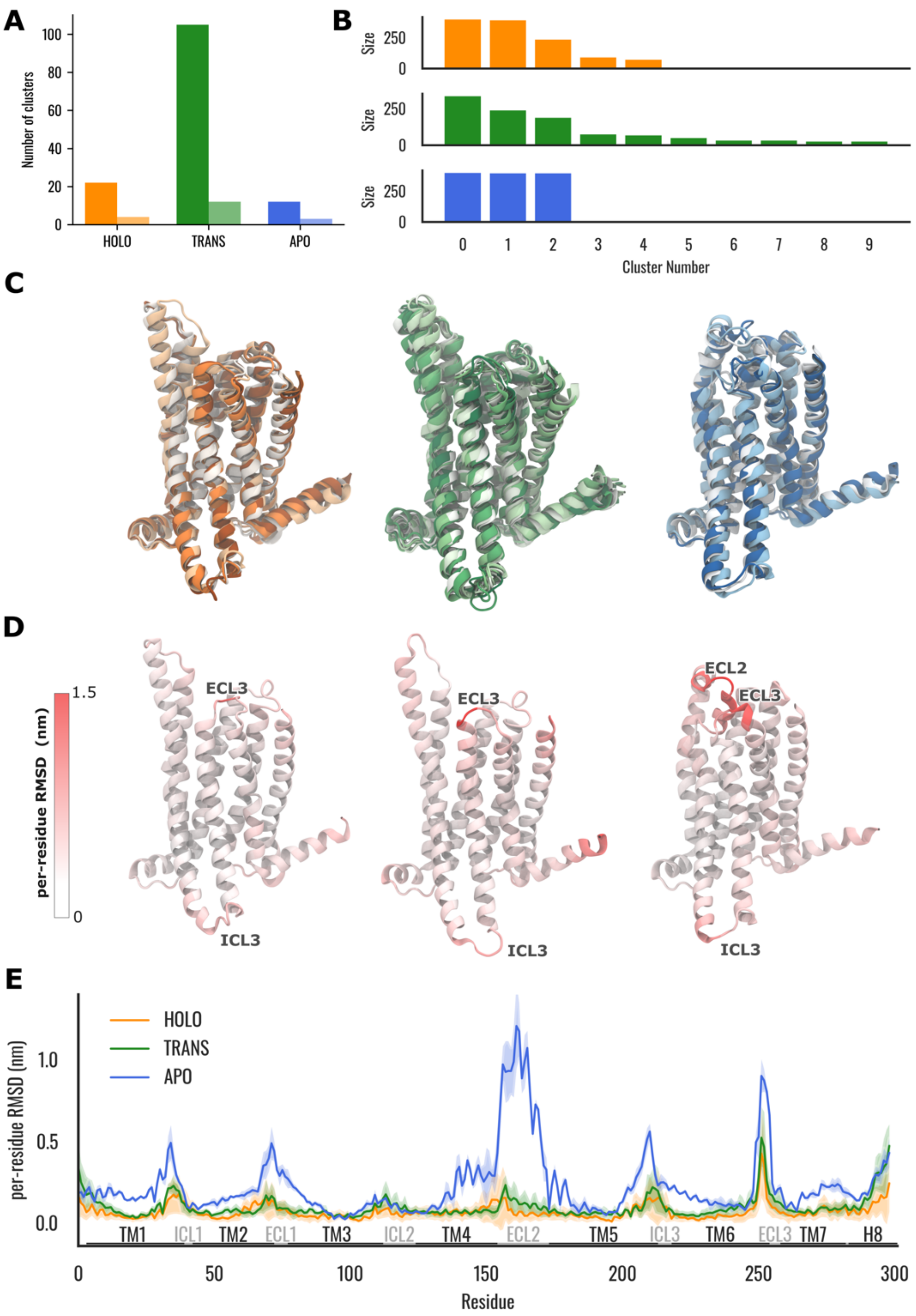

Supplementary Figure 5. Cluster analysis using the *single linkage* method implemented in GROMACS with an RMSD cutoff of 1 Å on the protein backbone over the concatenated 1.2 µs trajectory (last 400 ns of the three replicas with 1 ns time step) for each system. **(A)** Full colours represent the total number of clusters, while shaded colours show the number of clusters containing more than 90% of structures (trajectory frames). **(B)** Number of structures, i.e. the size, for the first 10 most populated clusters. **(C)** Visual representation of all centroids for clusters containing more than 90% of the structures. **(D)** Rendering of the centroids of the most populated clusters for each system. The reddish colour scale represents the average per-residue RMSD of all centroids compared to the centroid of the most populated cluster for the Holo state, which was used as a reference. Panel **(E)** represents the average per-residue RMSD values for the three systems.

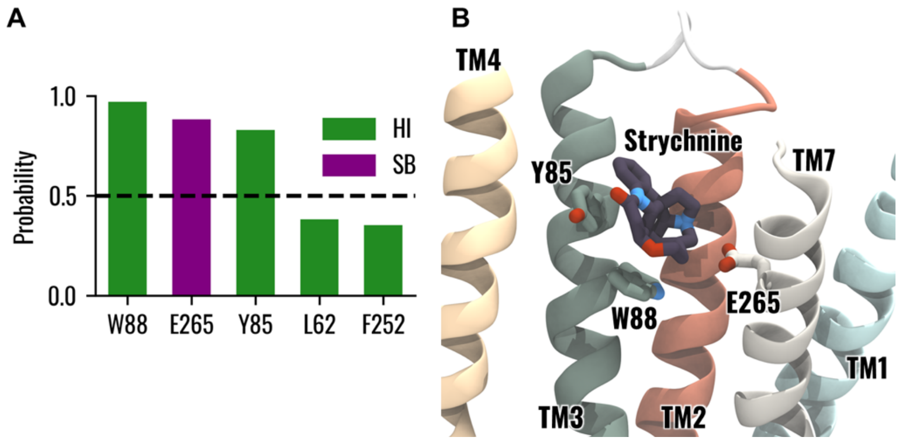

Supplementary Figure 6. (A) Probability of TAS2R46 residues to form an interaction with strychnine, as identified by PLIP software. The bars are coloured according to the specific type of interaction, i.e. HI=hydrophobic interaction (green), and SB=salt bridges (purple). (B) Visual representation of strychnine in TAS2R46 binding pocket. Residues forming interactions with a probability greater than 0.5 with Strychnine are shown.

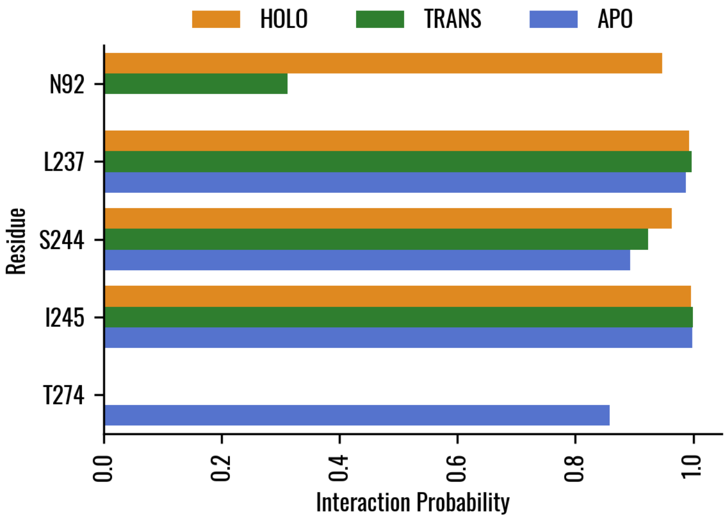

Supplementary Figure 7. Interaction probability between residues Y241$^{6.48}$ and the other residues of TAS2R46 during concatenated 1.2 μs trajectory (last 400 ns of the three replicas per system). The presence of an interaction between the residues was evaluated in each frame of the trajectory, sampled at a 2 ps time step. The total number of frames where an interaction was present was then divided by the total number of frames in the trajectory, resulting in an interaction probability value. The interactions with probabilities higher than 0.8 in at least one of the three systems (Holo, Trans, Apo) were all hydrogen bonds and are the only ones represented in the figure. The Holo state is represented in orange, the Trans state in green, and the Apo state in blue.

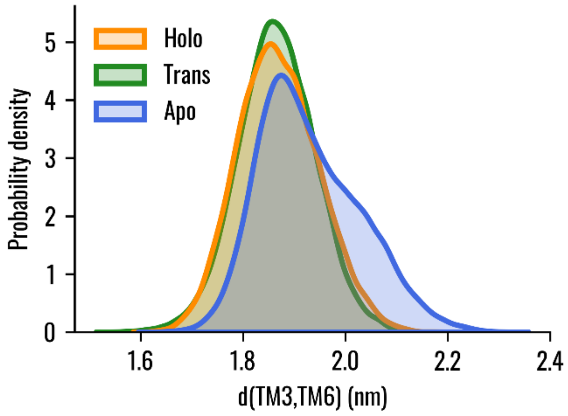

Supplementary Figure 8. Distribution of the distance between the intracellular areas of TM3 and TM6 in the three states, which has been used to characterize possible outward movement of TM6. The centre of mass of residues 104 to 100 has been considered to define the intracellular portion of TM3, whereas the centre of mass of residues 218 to 224 to define the intracellular area of TM6.

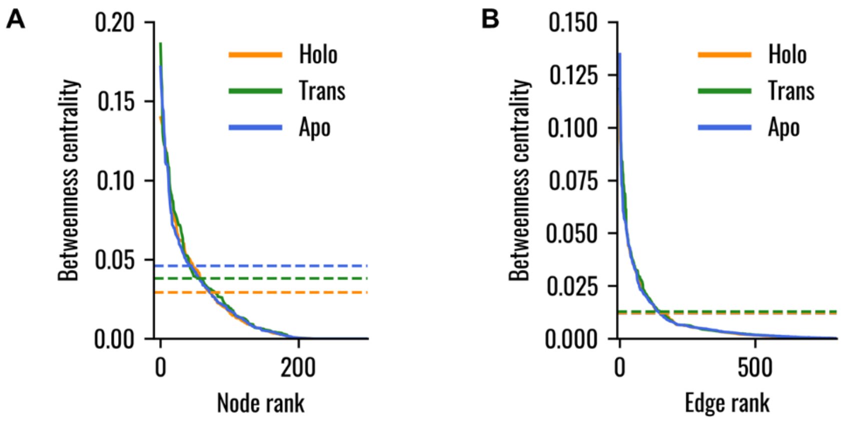

Supplementary Figure 9. Ranked betweenness centrality for the (A) nodes and (B) edges forming the dynamic networks of the Holo, Trans, and Apo systems. The horizontal dashed lines represent the value at which a knee was identified in the three systems. The identified threshold was the lowest knee identified.

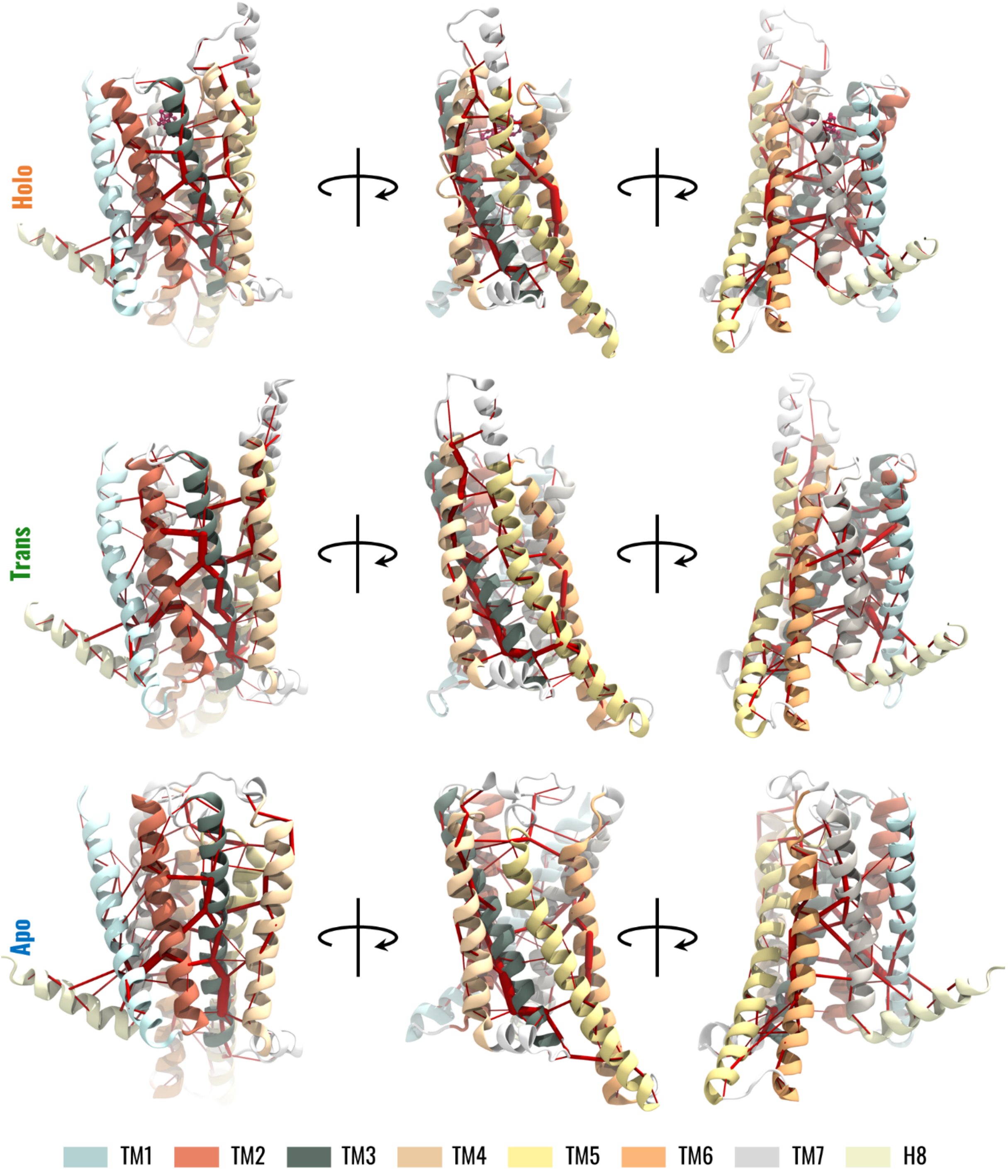

Supplementary Figure 10. Visual representation of the dynamic network for the three states of the receptor. The edges of the network are red cylinders with a radius proportional to the betweenness centrality of the edge.

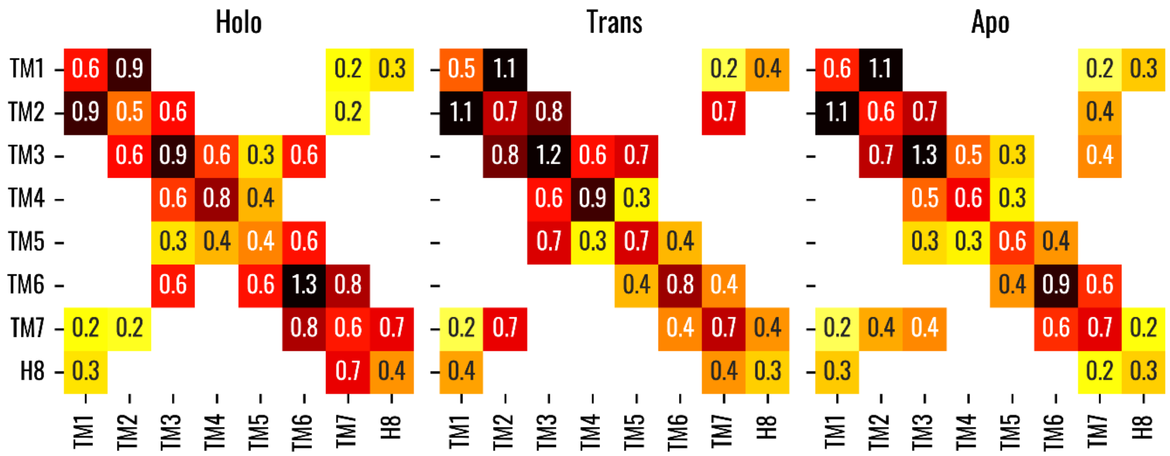

Supplementary Figure 11. Maximum betweenness of the edges linking different TM helices. Values represent the maximum betweenness, which has been multiplied by 10 for a better representation. White regions correspond to the absence of edges between the corresponding TM helices.

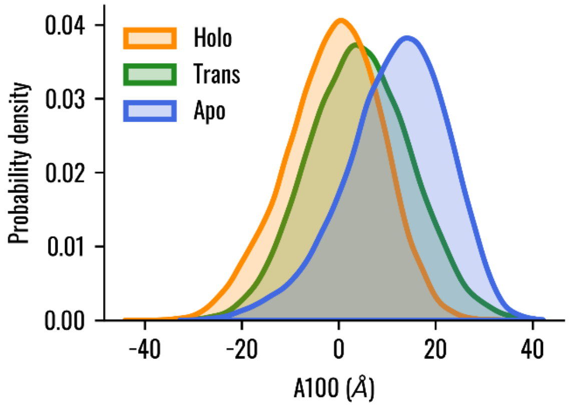

Supplementary Figure 12. Distribution of the A^{100} indicator in the Holo, Trans, and Apo states. It is worth mentioning that the change in the A100 index across the three systems demonstrated a different behaviour compared to what was proposed for class A GPCRs (Ibrahim et al., 2019).

3 Supplementary Tables

Supplementary Table 1. Summary of the optimal path connecting strychnine- and G protein-binding residues (visually represented in Figure 5 in the main text).

	**W88$^{3.32}$ – H224$^{6.31}$**	**E265$^{7.39}$ – Y106$^{3.50}$**
**APO**	**TM3 – TM5 – TM6**<br>[L107$^{3.51}$ → L197$^{5.60}$]<br>[L202$^{5.65}$ → A227$^{6.34}$]	**TM7 – TM3**<br>[Y271$^{7.45}$ – N96$^{3.40}$]
**TRANS**	**TM3 – TM5 – TM6**<br>[L107$^{3.51}$ → L197$^{5.60}$]<br>[L202$^{5.65}$ → H224$^{6.31}$]	**TM7 – TM2 – TM3**<br>[P272$^{7.46}$ – R55$^{2.50}$]<br>[L51$^{2.46}$ – L102$^{3.46}$]
**HOLO**	**TM3 – TM6**<br>[N92$^{3.36}$ – Y241$^{6.48}$]	**TM7 – TM6 – TM3**<br>[I267$^{7.41}$ – I240$^{6.47}$]<br>[Y241$^{6.48}$ – N92$^{3.36}$]

4 References

Calderón, J. C., Ibrahim, P., Gobbo, D., Gervasio, F. L., and Clark, T. (2023). General Metadynamics Protocol To Simulate Activation/Deactivation of Class A GPCRs: Proof of Principle for the Serotonin Receptor. *J. Chem. Inf. Model.* 63, 3105–3117. doi: 10.1021/acs.jcim.3c00208

Ibrahim, P., Wifling, D., and Clark, T. (2019). Universal Activation Index for Class A GPCRs. *J. Chem. Inf. Model.* 59, 3938–3945. doi: 10.1021/acs.jcim.9b00604

Zhou, Q., Yang, D., Wu, M., Guo, Y., Guo, W., Zhong, L., et al. (2019). Common activation mechanism of class A GPCRs. *eLife* 8, e50279. doi: 10.7554/eLife.50279
